# National Cancer Control Program of Thailand 

**DOI:** 10.31557/APJCP.2020.21.3.577

**Published:** 2020-03

**Authors:** Weerawut Insamran, Suleeporn Sangrajrang

**Affiliations:** *National Cancer Institute, Ratchathewi Road, Bangkok, Thailand. *

**Keywords:** Cancer control program, capacity, research training, cooperation, Thailand

## Abstract

Since 2000 cancer has been the leading cause of death in Thailand. In response to this challenge, the National Cancer Institute of Thailand (NCI), in collaboration with other bodies, has developed and promoted the National Cancer Control Program (NCCP) to provide appropriate policies and practice for the prevention, early detection and treatment of cancer, with optimal supportive care. With plans strongly supported by the Ministry of Public Health, the NCCP envisages integration into the health care system in 6 strategic areas: (1) cancer informatics; (2) primary prevention; (3) early detection; (4) treatment; (5) palliative care; and (6) cancer control research. For this purpose 7 regional cancer hospitals have been established to aid the NCI in conducting the NCCP. Cancer registration is a high priority, with 31.2% of the population now covered by quality registries. In primary prevention, there is a focus on awareness, lifestyle improvement, anti-smoking and alcohol control programs, vaccination, and *Opisthorchis viverrini *(OV) control. Screening programs for cervical, breast and colorectal cancer are underway to increase early detection. Priority is being given to facilities for chemotherapy and radiotherapy, as well as palliative care. Cancer control research encompasses international cooperation and participation in training programs, especially for development of cancer registration and other aspects of cancer control programs in South-East Asia, not least as an IARC Collaborating Center.

## Introduction

**Figure 1:**
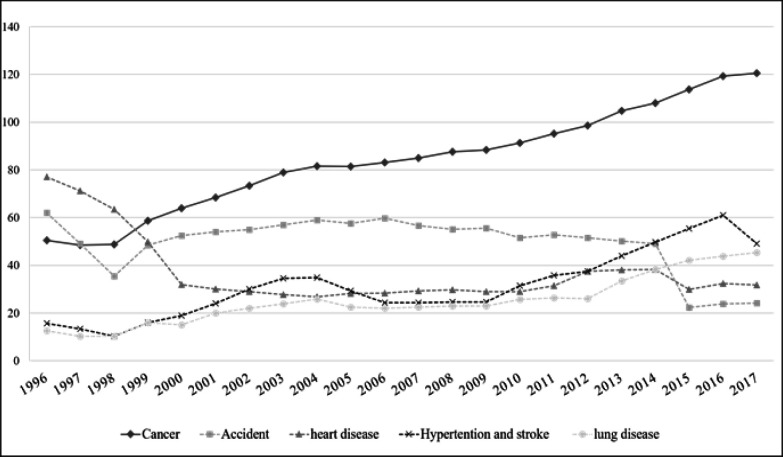
Mortality Rates Per 100,000 Population of 5 Major Causes of Death in Thailand

During the last 20 years, the leading cause of death in Thailand has changed from infectious to non-communicable diseases like cardiac and lung disease, hypertension, cerebrovascular disease, and cancer. Rates for malignant neoplasms have gradually increased over the past decade and since the year of 2000, cancer has been the leading cause of death in Thailand (Figure 1). 

In response to the challenge of cancer in a manner consistent with World Health Organization guidelines, the National Cancer Institute of Thailand (NCI), in collaboration with other bodies, has developed and promoted the National Cancer Control Program (NCCP). The NCCP provides appropriate policies and practice for the prevention, early detection and treatment of cancer, with optimal supportive care, five-year plans were developed at each NCCP meeting (1997, 2013 and 2018). Attendees consisted of stakeholders from the public and private sector and NGOs. The Ministry of Public Health has strongly supported the NCCP integration into the health care system in 6 strategic areas: cancer informatics; primary prevention; early detection; treatment; palliative care; and cancer control research. 

**Figure 2:**
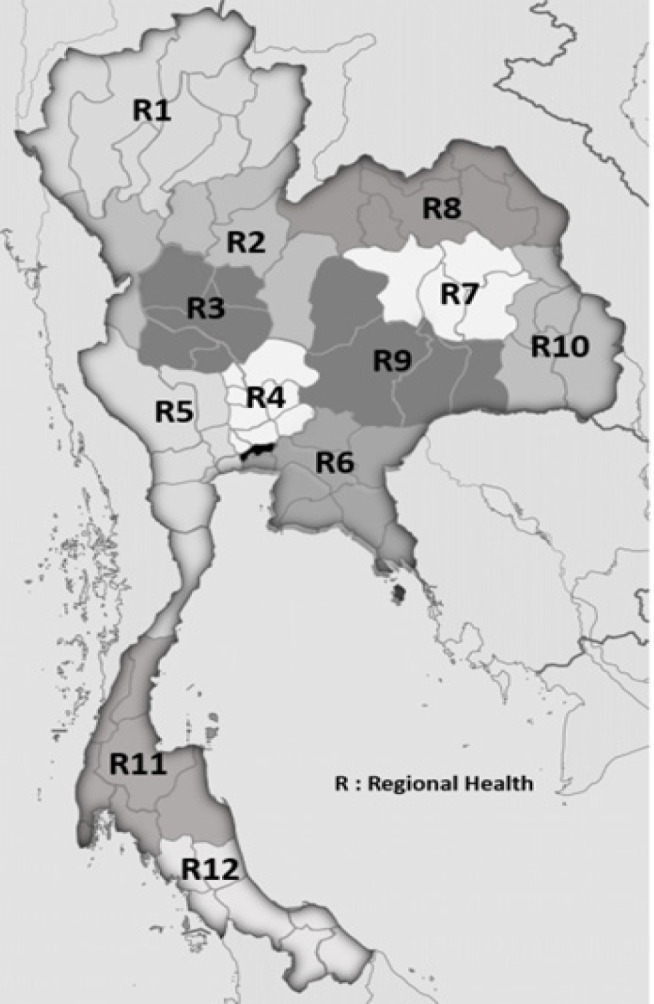
Regional Health Areas and Regional Cancer Hospitals

Under the reform movement of the Ministry of Public Health, a service plan was implemented in 2013, when 12 regional health areas and Bangkok were assigned as pilot sites for the NCCP (Figure 2). The NCI and regional cancer hospitals were directed to support implementation of cancer control activities. 

*Cancer Informatics*

The cancer registry is an essential part of any national programme for cancer control. Accurate data from both population-based and hospital-based registries are needed for planning and monitoring cancer control strategies and for identifying priorities in public health. 

In Thailand, the National Cancer Institute began cancer registration in 1971 with the collection of information on cancer patients treated in 53 hospitals throughout the country. The first Population-Based Cancer Registry (PBCR) was launched in 1986 in Chiang Mai, followed by Khon Kaen in 1988, Songkhla and Bangkok in 1990 and Lampang in 1993. The International Agency for Research on Cancer (IARC), and the registries in Thailand published the first volume of Cancer in Thailand in 1993 (Vatanasapt et al., 1993). The second volume followed in 1999 (with data for 1992-1994), and the third in 2003 (Deerasamee et al.,1999; Sriprung et al., 2003). Further updates have been published regularly (Khuhaprema et al., 2007; 2010; 2012b; 2013; Imsamran et al., 2015; 2018).

**Figure 3:**
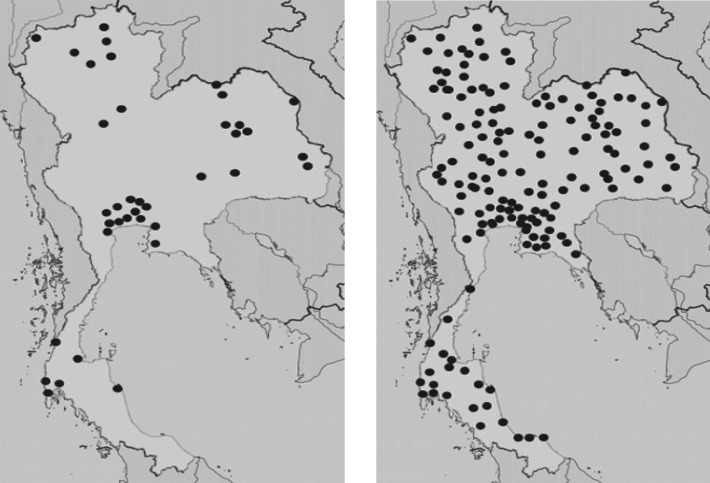
Hospital Based Cancer Registry 2014 and 2018

Currently there are 15 PBCR’s in Thailand. Data from 11 of these were included in the last volume of Cancer in Thailand which covered about 31.2% of the population. The Ministry of Public Health had proposed that every provincial hospital should develop a hospital-based cancer registry (HBCR). By 2014, 162 hospitals in 43 provinces, and in 2018, 580 hospitals in 77 provinces had done so (Figure 3). This is expected to achieve considerable improvement in the quality of cancer registry data.

**Figure 4:**
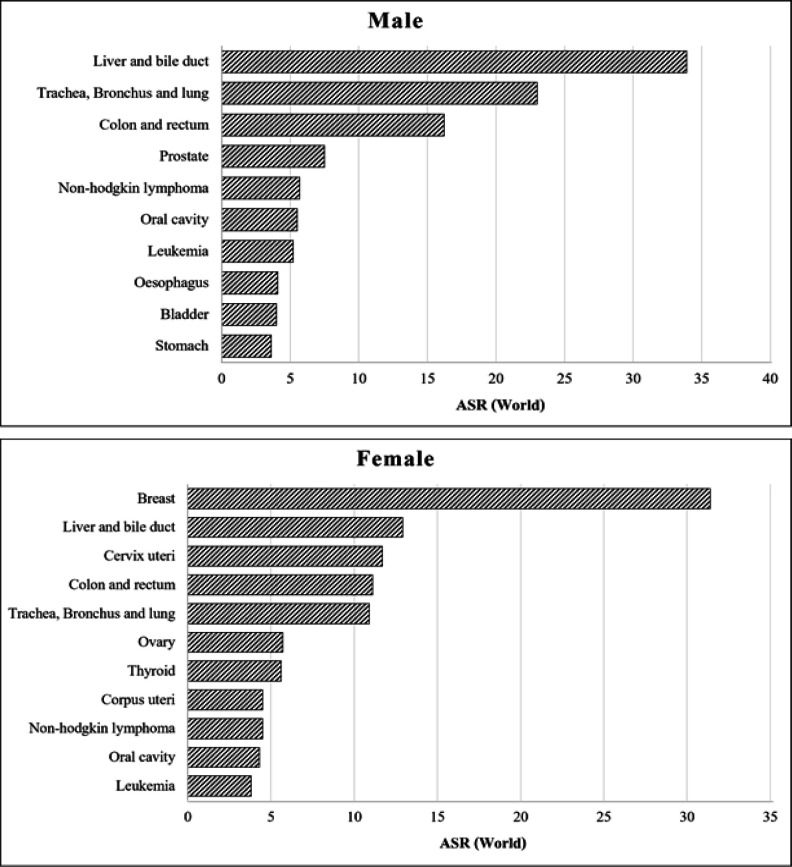
Leading Cancers in Thailand 2013-2015

The estimated number of new cancer cases in Thailand in 2014 was 59,662 in men and 63,095 in women, corresponding to age-standardized rates of 143.8 and 134.2 per 100,000 respectively (Table 1). From national estimates, the five leading cancer types are: liver, lung, colorectal, prostate cancer and Non-Hodgkin lymphoma among men; and breast, liver, cervical, colorectal and lung cancer among women (Figure 4).

**Figure 5:**
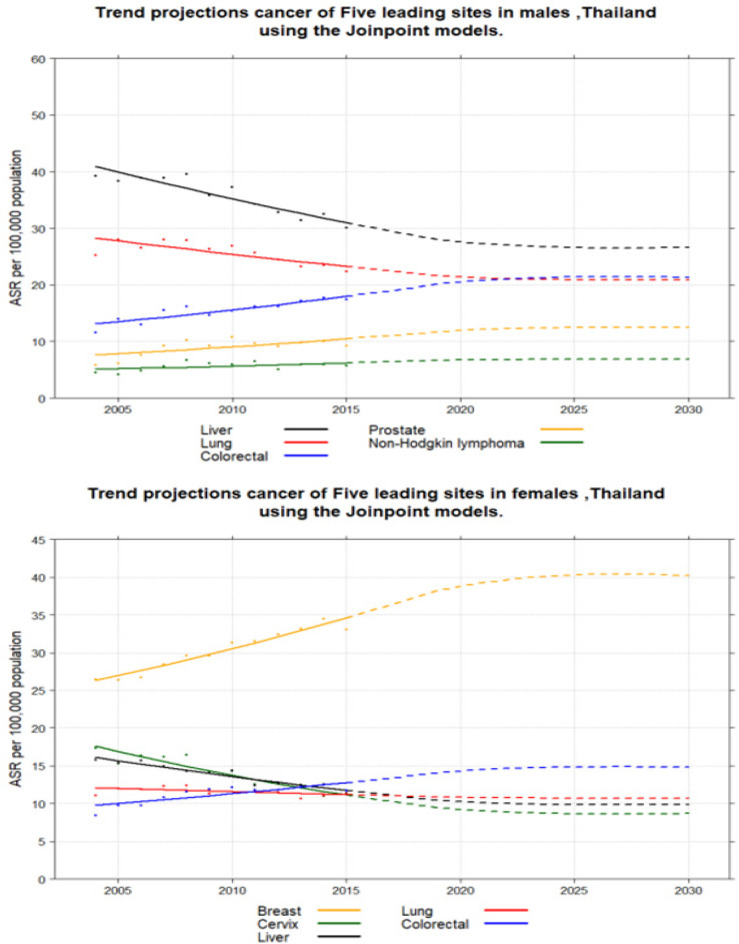
Trends Over Time for Leading Cancers in Thailand

We projected cancer incidence at the National for the various calendar years from 2004-2015 to 2025 at 5-yearly intervals (Figure 5). Trends by age-adjusted incidence, the Joinpoint Regression Analysis program, version 4.6.0.0, was used. Joinpoint fits a linear regression model to the data to detect when statistically significant changes in the trend occur. A significance level of 0.05 was used for the permutation test which determines the minimum number of “joinpoints” necessary to fit the data.

For 2025, 158,785 new cancer cases have been predicted, 75,974 in males and 82,811 in females. The incidence of liver cancer is expected to continuously decrease in both sexes, with an expected number of 24,616 new cases detected overall in 2025 for the whole country, 16,962 in males and 7,654 in females. Lung cancer incidence is also expected to decrease in both sexes to 12,350 male and 7,560 female cases in 2025. Colorectal cancer incidence is increasing, with over 19,064 new cases expected in 2025, comprising10,335 male and 8,729 female. In women, breast cancer is rapidly increasing, with over 19,452 new cases expected in 2025 (Imsamran et al., 2018). Fifty-five percent of cases were found to involve pre-menopausal women in one study in Thailand (Ekpanyaskul et al., 2010). In contrast, cervical cancer numbers are sharply decreasing with around 4,352 new cases expected in 2025. The odds of developing cervical cancer declined in all provinces (Thongsak et al., 2016). A decrease was projected in all geographic zones except the North-East during 2012-2025 (Virani et al., 2017). However, a decreasing trend in incidence of cervical cancer in Khon Kaen was evident from 1990 to 2014 with a prediction of continuous decrease until 2029 (Saenrueang et al., 2019). Cervical cancer incidence in Songkhla peaked around 1998-2000 and then dropped by 4.7% per year (Sriplung et al., 2014). 

**Primary Prevention**

In the field of cancer prevention, many activities are being promoted in Thailand, as follows: 

*Vaccination*

Hepatitis B vaccination for newborn babies was introduced as part of Thailand’s Expanded Program Immunization (EPI) in 1992. The sero positivity rates of HBsAg pre and post-EPI were 4.5 and 0.6%, respectively (Posuwanet al., 2016).

Since 2017, implementation of HPV vaccination has been proposed as part of the national program. The protocol comprises2 doses of HPV vaccine given to female students in grade 5 aged 9-11 years. The Department of Disease Control has reported a vaccine coverage of 95% (around 400,000 girls) with no severe adverse effects.

*Anti – smoking campaign*

Thailand has a strong anti-smoking program. The Non–Smokers Health Protection Act was introduced in 1992, and in 1997, the Thai parliament passed a bill which further strengthened Thailand’s commitment to the provision of smoke–free areas. There is a complete ban on smoking in air–conditioned premises, along with a total ban on advertising and sponsorship. The program features:

• Notification of the composition of tobacco products. 

• Vending machines for the sale of cigarettes are no longer permitted. 

• Health warnings must be prominent on packaging. 

• Sale to minors is prohibited.

• A Quitline service which has operated since 1999. 

• Smoking cessation clinics in Public Health Centers across the nation to help people who want to quit smoking. 

*Alcohol consumption Control*

The Office of Alcohol Beverages has been established by the Ministry of Public Health. The Alcohol Beverage Control Act dictates regulations and campaigns to reduce the consumption of alcoholic beverages. 

*OV control*

Cholangiocarcinoma is still one of the most common cancers in Thailand, especially in the Northeastern region. It has been known for a long time that eating raw fish harboring the infective stage of the OV liver fluke is the cause. Campaigns for consumption of cooked food have been conducted by the Ministry of Public Health for over 30 years. While the prevalence of OV infection has been shown to be declining over time, the incidence of cholangiocarcinoma remains relatively high. We therefore continue to focus on prevention, training our primary health care workers how to change attitudes and beliefs about cooking in local communities.

*Healthy life-style promotion*

A healthy diet is essential to help prevent cancer. NCI therefore launched the slogan “half veggie a meal” to make it easy for people who follow recommendations. The Ministry of Public Health has also conducted many programs to promote physical activity, exercise and avoidance of obesity, using the slogan “no more belly fat”. Since 2006, NCI has played a leading role in carrying out cancer prevention campaigns, with five does and don’t using a wide variety of education materials, talks at schools, and public education through all social media including TV, radio, newspapers and internet.

**Early Detection**

Increasing awareness of the signs and symptoms of cancer contributes to the early detection of disease. Where tests for cancer of specific sites are available, screening of apparently healthy individuals can disclose cancer in precursor or early stages, when treatment will be most effective.

*Screening Programs in Thailand*

1. Program for screening of cervical cancer: Pap smear aged 30-60 every 5 years 

2. Program for screening of breast cancer: BSE aged 30-70 years, CBE aged 40-70 years with mammography as an opportunistic approach

3. Program for screening of colorectal cancer: Fecal Immunochemical Test (FIT) aged 50-70 years every 2 years.

*Cervical cancer*

At present, we have a national policy to perform Pap smear tests in women aged 30-60 years at 5-year intervals free of charge. Screening is generally conducted in primary health care centers and hospitals. In addition, in some areas Visual Inspection Acetic Acid (VIA) is available for women aged 30-45 years. Each year, around 1,000,000 women are screened for cervical cancer. With the organized Pap smear cervical cytology screening in Thailand, between 2005 and 2009, 69.2% of the 4,030,833 targeted women were screened, but information on the management of precancerous lesions was available for only 17.4% of women referred for colposcopy (Khuhaprema et al., 2012a). The referral rate for colposcopy in women with equivocal lesions is high and since loss to follow-up is a major limitation, immediate colposcopy should be offered for women at high risk (Perksanusak et al., 2015). During 2010-2014, 5,164,751 women were screened by pap smear. We have investigated the follow up of abnormal women in 4 provinces and found that around 70% had been referred for further investigation (Data not shown).

It is reported that the hrHPV test is superior to cytology for the early detection of high-grade cervical epithelial lesions (Cuzick et al., 2008). Our pilot study in Ubon Ratchathani demonstration the feasibility of integrating HPV testing into public health services in Thailand (Sangrajrang et al., 2017). In addition, cost-effectiveness analysis provided support for full implementation of HPV testing as the primary method for cervical screening in Thailand (Termrungruanglert et al., 2017). In 2020, the Ministry of Public Health plan to scale up the program throughout the country.

*Breast Cancer*

Breast self-examination (BSE) and clinical breast examination (CBE) are promoted to enhance public awareness of breast cancer. Women aged over 45 years are also encouraged to undergo a mammography check-up every 2 years, although our cancer centers only provide this on an opportunistic basis. A new X-ray mammography mobile unit is expected to increase uptake in the country. 

*Colorectal cancer*

After a pilot study of colorectal screening using immunochemical test to test feasibility, acceptability and cost effectiveness of a population-based program (Khuhaprema et al., 2014), a nation-wide program for the population aged 50-70 years was launched in 2018. Screening is conducted in Primary Health Care using Faecal Immunochemical tests (FIT), and positive cases receive further investigation by colonoscopy.

**Treatment**

Our cancer care teams work together at national and international level towards improving cancer treatment. To gain more treatment coverage, seven regional cancer centers were introduced in 1989. 

Thailand has provided universal health coverage since 2002, with three major national health insurance schemes, i.e., the Universal Health Coverage Scheme (covering 48 million); Social Health Insurance (covering 10.6 million); and the Civil Servant Medical Benefit Scheme (covering 4.4 million) (Tangcharoensathien et al., 2018). All include access to cancer treatment.

Following the service plan policy, each health area has been tasked with building capacity for cancer care and treatment including improvement of the referral system. Currently, 34 advanced and 51 standard hospitals provide surgery and chemotherapy services. All 13 health areas have at least one radiotherapy center and the Ministry of Public Health plans to establish more radiotherapy capacity in areas of need.

**Palliative Care**

Palliative care was one important arm of the strategic plan for the NCCP 1997-2001. The NCCP for 2013-2017 reaffirmed palliative care development, in compliance with WHO Guidelines for effective programs: Cancer Control, Knowledge into Action, Palliative Care. The Department of Medical Services of the Ministry of Public Health is responsible as the key mover of this policy initiative at the national level, with support from academic institutions, Practical guidelines have been developed and currently, palliative care units are active in almost all general and community hospitals.

**Cancer Research and International Cooperation**

The goal of cancer control research is to identify and evaluate means of: reducing cancer morbidity and mortality; and improving the quality of life of cancer patients and their families. Research is a key component in the development, implementation and evaluation of national cancer control programs. Our research encompasses many areas and disciplines, including basic, clinical epidemiological and translational efforts. Over the last few decades, many collaborative research projects have been conducted between our scientists and experts from leading cancer research institutes in the region. In particular cooperation with IARC and the Global Initiative for Cancer Registration has resulted in establishment of a Collaborating Center to facilitate holding training courses and meetings. However, almost all research projects are at the individual or institute level, and Thailand still lacks the funding it needs and the research directions for cancer control.

**Table 1:** Cancer Incidence in Thailand

		1990	1993	1996	1999	2002	2005	2008	2011	2014
Male	Number	29,950	32,801	35,539	31,582	40,662	48,596	50,961	54,586	59,662
	ASR	153.6	151.3	149.2	127.7	143.3	158.8	156.7	143.3	143.84
Female	Number	29,517	30,940	38,467	33,678	39,688	50,256	51,825	57,806	63,095
	ASR	128.5	123.8	125	125.5	118.6	138.3	138.2	131.9	134.15
